# Novel Suturing Methods for the Management of Traumatic Choroidal Avulsion in Globe Injuries

**DOI:** 10.3389/fmed.2021.801068

**Published:** 2022-01-17

**Authors:** Huijin Chen, Jiarui Yang, Changguan Wang, Xuefeng Feng, Kang Feng, Zhizhong Ma

**Affiliations:** ^1^Department of Ophthalmology, Peking University Third Hospital, Beijing, China; ^2^Beijing Key Laboratory of Restoration of Damaged Ocular Nerve, Beijing, China

**Keywords:** choroidal avulsion, ocular trauma, trans-scleral mattress suturing, intraocular suturing, no light perception

## Abstract

**Purpose:**

To explore the long-term efficacy of novel choroidal suturing methods including trans-scleral mattress suturing (TSS) and intraocular suturing (IOS) in the treatment of choroidal avulsion.

**Design:**

Prospective cohort, hospital-based study.

**Methods:**

A total of 24 patients who were diagnosed with choroidal avulsion were enrolled in this study. The demographic characteristics, baseline information of trauma, best-corrected visual acuity (BCVA), and intraocular pressure (IOP) were collected before surgery, and the anatomic abnormities of the globe were recorded before or during surgery. All patients were diagnosed with choroidal avulsion and underwent choroid suturing treatment during vitrectomy, postoperative functional variables including BCVA and IOP, anatomic variables including retinal and choroidal reattachment rate, and silicone oil migration rate, which were recorded at the regular follow-ups at least 1 year after surgery.

**Results:**

All patients with open globe injury involved zone III, 70.8% of the patients presented with two quadrants of the avulsed choroid, and 29.2% with one quadrant involved; moreover, all patients had complications with retinal detachment (RD), of which 58.3% of patients had closed funnel retinal detachment. TSS was applied in nineteen patients and IOS in five patients. Postoperatively, a significant improvement on LogMAR BCVA was observed at each follow-up from 3.57 ± 0.69 before surgery to 2.82 ± 0.98 at the last follow-up (*p* < 0.05), and the proportion of no light perception (NLP) was also reduced from 69.6 to 37.5%. IOP was markedly elevated from 6.4 ± 4.1 mmHg preoperatively to 11.3 ± 4.3 mmHg at the last follow-up (*p* < 0.05). Choroidal reattachment was achieved in 91.7% of patients; two patients were observed with silicone oil migration at 3 months after surgery and underwent drainage of suprachoroidal silicone oil and sclera buckling. Meanwhile, retinal attachment was observed in 95.8% of patients, only one patient developed partial RD due to postoperative proliferative vitreoretinopathy, and secondary vitrectomy was performed; all patients were observed with complete retinal and choroidal attachment at the last follow-up. Eventually, four patients were silicone oil-free, and 20 patients were silicone oil-dependent.

**Conclusions:**

Choroidal suturing proved to be an effective method to fix the avulsed choroid, which greatly improved the BCVA and maintained the IOP, and efficiently increased the choroidal and retinal reattachment rate and preservation of the eyeball.

## Introduction

Ocular trauma remains one of the major causes of visual impairment and even blindness in the working population worldwide, especially when the posterior segment is involved (1, 2). In addition to retinal injuries, traumatic choroidal injuries are also severe complications and are closely associated with the prognosis of ocular trauma. It was reported that choroidal injuries were observed in 69.7% of all the eyes with no light perception and were estimated as a predictor of poor prognosis (3). Traumatic choroidal injuries are not uncommon in clinical practice, and a variety of choroidal injuries have been reported (4–7). In our recent study (8), we summarized traumatic choroidal injuries into nine categories, including suprachoroidal effusion, suprachoroidal hemorrhage, massive suprachoroidal hemorrhage, choroidal avulsion, traumatic chorioretinal rupture, choroidal rupture, choroidal loss, choroidal hole, and choroidal damage at the wound site, and a classification system for traumatic choroidal injuries was proposed. Among the nine categories, choroidal avulsion was one of the most catastrophic, and it was found that 92.2% of eyes with choroidal avulsion had unfavorable outcomes (8).

The concept of choroidal avulsion was first brought up by *Bordeianu* in 1984, and we have recently redefined the term as a sudden severe hemorrhagic separation of the choroid from the sclera, accompanied by discontinuity of the detached choroid or, sometimes, choroid and ciliary body avulsed at scleral spur as a whole unit (8, 9). It has been nearly half a century since the concept was raised; however, literature focusing on choroidal avulsion is scant, mainly because of the lack of effective treatment. Despite the development and refinement of vitreoretinal microsurgical techniques, eyes with choroidal avulsion can hardly be repaired. They are almost always complicated with persistent hypotony, which results from the connection of the vitreous cavity and the suprachoroidal cavity after uveal injuries, leading to the free flow of the aqueous humor and all intraocular tamponade between these two compartments, which lowers the intraocular pressure. Moreover, the formation of the two compartments significantly reduces the volume of the vitreous cavity, which results in the decreased possibilities of retinal reattachment. Therefore, without effective treatment, eyes with choroidal avulsion usually end in phthisis bulbi and cannot be preserved.

Surgical treatment of choroidal avulsion should be full of challenges. Very few surgical techniques have been reported in the literature (6, 7). In the present study, novel suturing methods including trans-scleral mattress suturing (TSS) and intraocular suturing (IOS) were developed to refix the avulsed choroid and the postoperative conditions were evaluated.

## Subjects and Methods

### Study Design and Participants

This study was a prospective cohort study conducted following the principles of the Declaration of Helsinki and was approved by the Human Research and Ethics Committee of Peking University Third Hospital. All information was collected from the database of Eye Injury Vitrectomy Study (EIVS), which has been a hospital-based multicenter, prospective cohort study since January 1997. Patients in the present study were recruited from the Department of Ophthalmology at Peking University Third Hospital, consecutively, between April 2013 and July 2017. Informed consent was obtained from all patients or their direct relatives if the patient was under 18.

The study included patients who were diagnosed with choroidal avulsion in the surgery and received TSS or IOS refixation of the avulsed choroid suturing treatment. Since nearly all cases with choroidal avulsion were complicated with severe intraocular hemorrhage, it was difficult to identify the choroidal avulsion before the surgery. The decisions of suturing were made intraoperatively. All cases with choroidal avulsion found during surgery were sutured unless the extension of choroidal avulsion was >2 quadrants, and/or there was total retinal loss. For this study, patients with the following conditions were excluded including primary ocular diseases other than refractive errors and cataract (especially uveitis or other inflammatory diseases, which might influence the function of the choroid); intraocular surgeries before ocular injury; patients over 80; and patients without regular follow-ups for half a year. Eventually, 24 patients were enrolled in this study.

### Clinical Evaluation

The clinical evaluation of all the enrolled patients was performed in the following order: demographic information (including age, sex), baseline information of trauma (including injured eye, type of injury, time interval from trauma to the primary surgery, time interval from trauma to the vitrectomy), and routine ophthalmic examinations (including best-corrected visual acuity (BCVA), intraocular pressure (IOP), and intraocular complications of the injured eye, which was assessed during exploratory vitrectomy surgery by two chief surgeons (MZZ and CHJ). The evaluation procedure was carried out following a standardized “Register of Eye Injury” form as reported in the previous study (10). The VA was converted to LogMAR for analysis. Counting fingers at 1-meter vision was converted to 1.87 LogMAR, Hand motions vision to 2.3 LogMAR, and light perception vision to 2.8 LogMAR, for those with no light perception, 4. LogMAR was applied (11).

Postoperative follow-ups were regularly performed, and BCVA, IOP, retinal and choroidal reattachment rates, percentage of the silicone oil migration to suprachoroidal cavity, and postoperative complications were assessed at each follow-up. Subsequent treatment was applied according to the clinical findings at each follow-up.

### Surgical Procedure

**Figure 1:**
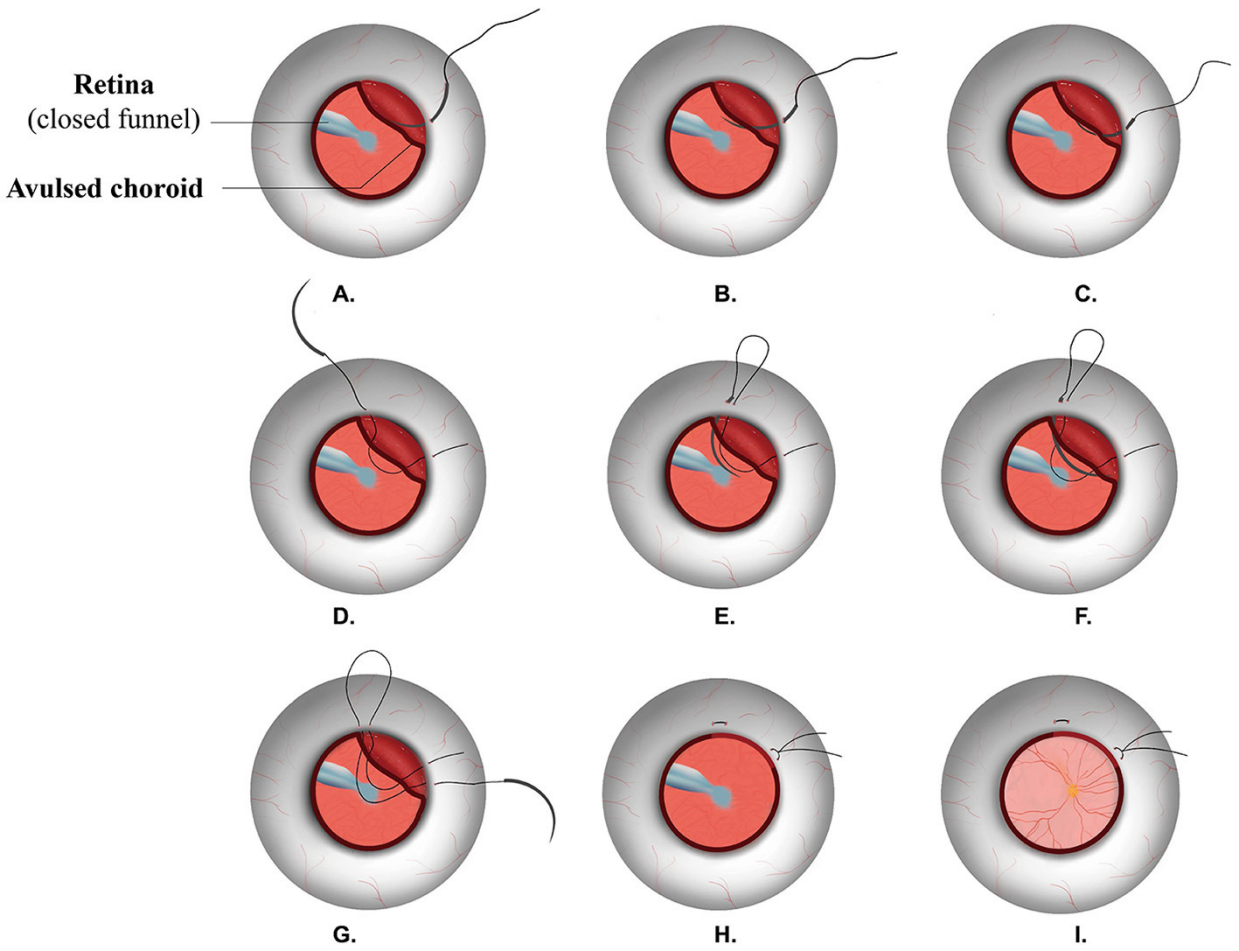
Surgical procedures of TSS. TSS: trans-scleral mattress suturing. A long-curved needle with a 10-0 polypropylene suture **(A)** was inserted at the sclera 6 mm posterior to the limbus, **(B,C)** passed through the avulsed choroid, **(D)** exited from the sclera, **(E)** a U-turn was made, and the needle was reinserted near the exit site; **(F,G)** the suturing maneuver was repeated, passing through near the insertion site, **(H)** a knot was tied, **(I)** a retinal funnel was unfolded, and retina was reattached.

All patients received a standard 3-port 20-gauge pars plana vitrectomy under general anesthesia. After the removal of intraocular hemorrhage and other opacities in ocular media, the extension of the avulsed choroid was assessed, and the corresponding location was marked on the sclera. Suturing techniques, TSS or IOS, were applied to reattach the avulsed choroid to the sclera. IOS was chosen if the anterior part of the avulsed choroid was missing and/or the choroid was highly detached, making the suturing unreachable from the outside of the eye. The description of the two suturing techniques is as follows:

For patients receiving TSS (Figure 1), a long-curved needle with a 10-0 polypropylene suture (Alcon laboratories, Inc. 8065307901) was inserted into the eye at the sclera 6 mm posterior to the limbus, passed through the avulsed choroid, exited from the sclera, approximately 8- to 10-mm wide apart from the insertion site, and then a U-turn was made, the needle was reinserted near the exit site, and the suturing maneuver was repeated, passing through near the insertion site, and a knot was made. The passing route of the suture paralleled with the limbus. Several pairs of sutures were made to cover the extent of the choroidal avulsion.

**Figure 2:**
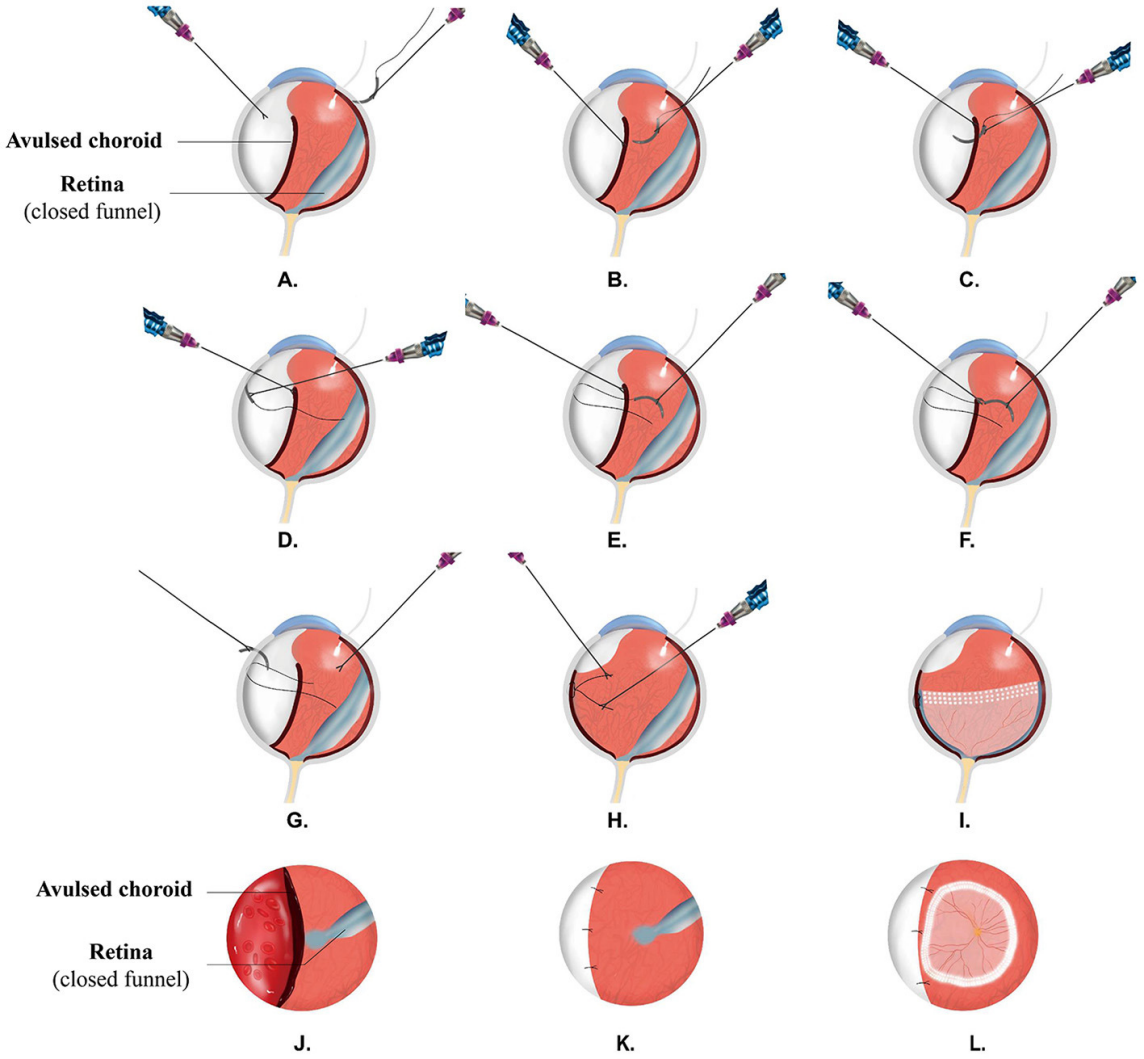
Surgical procedures of IOS. IOS: intraocular suturing. **(A-I)** presents the sideview. Chandelier illumination was used to assist bimanual manipulation, **(A)** a needle with 8-0 polyglactin suture was introduced into the vitreous cavity through the right port of 20-G sclerotomy, **(B)** two intraocular forceps were inserted, with the right forceps holding the needle and the left one capturing the anterior margin of the avulsed choroid to accomplish the suturing. The needle passed through the full-thickness choroid **(C)**, lamellar sclera **(D)**, and through the choroid again **(E)**; suture at the needle side was cut **(F)**, and a needle was taken out of the eye **(G)** before a knot was tied **(H)** by the two intraocular forceps, **(I)** a retinal funnel was unfolded, and retina was reattached. **(J-L)** presents the front view. **(J)** Showing avulsed choroid with closed funnel retinal detachment, **(K)** the avulsed choroid was refixed to the sclera with intraocular-interrupted suturing, and the connection of vitreous cavity and suprachoroidal cavity was blocked, **(L)** the retinal funnel was unfolded, and retina was reattached.

For patients undergoing IOS (Figure 2), chandelier illumination was used to assist bimanual manipulation. A needle with 8-0 polyglactin 910 suture (ETHICON, LLC. EMW9560) was introduced into the vitreous cavity through the right port of 20G sclerotomy and was placed in the center of the vitreous cavity; two intraocular forceps (MR-G113T, Suzhou Mingren Medical Equipment Co., Ltd., China) were inserted through the sclerotomy on both sides, with the right forceps holding the needle and the left one capturing the anterior margin of the avulsed choroid to accomplish the suturing. The needle passed through the full-thickness choroid, lamellar sclera, and through the choroid again, trying to minimize sliding of the suture through the choroid, which can cause a cutting effect on the choroid. Then, the suture at the needle side was cut, and a needle was taken out of the eye before a knot was made by the two intraocular forceps. For each stitch, a new needle with a suture was used. Usually, three stitches were necessary to cover a 180° avulsion.

After the repair of the choroidal avulsion, the retinal funnel was unfolded, and the retina was reattached by additional procedures such as epiretinal or subretinal membrane peeling, retinotomy and retinectomy, use of perfluorocarbon liquid (PFCL), retinal photocoagulation, and silicon oil or gas tamponade.

### Statistical Analyses

Statistical analyses were performed using SPSS software version 22.0 (SPSS, Inc., Chicago, IL). For each variable, grouped and overall analyses were conducted. Basic characteristics in all enrolled patients were described using descriptive statistics including means ± standard deviations for continuous variables, and frequencies (proportions) for categorical variables. Paired *t*-test was used to evaluate the differences of BCVA, IOP at follow-ups from the baseline. A *P* < 0.05 was considered statistically significant.

## Results

### Demographic and Preoperative Information

A total of 24 patients' eyes with choroidal avulsion (aged from 5 to 72) who underwent vitrectomy with choroidal suturing at Peking University Third Hospital between the period of April 2013 and July 2017 were enrolled, of which 19 patients underwent TSS and 5 patients with IOS. All patients finished the 1-year follow-up, and the mean overall follow-ups were 460 ± 83 days. Demographic and preoperative information, including age, sex, types of injury, and disease progression, is shown in Table 1.

**Table 1:** Basic characteristics of enrolled subjects.

**Objectives**	**ALL**	**TSS**	**IOS**
	**(*n* = 24)**	**(*n* = 19)**	**(*n* = 5)**
Age, mean ± SD	40.8 ± 15.0	40.8 ± 15.6	40.6 ± 9.1
Sex, *n* (%)			
Male	16 (66.7%)	11 (57.9%)	5 (100.0%)
Female	8 (33.3%)	8 (42.1%)	0 (0%)
Type of injury, *n* (%)			
Rupture	22 (91.7%)	17 (89.5%)	5 (100.0%)
Penetrating	1 (4.2%)	1 (5.3%)	0 (0%)
Contusion	1 (4.2%)	1 (5.3%)	0 (0%)
Traumatic zone, n (%)			
I/ II	0 (0%)	0 (0%)	0 (0%)
III	23 (100.0%)	18 (100.0%)	5 (100.0%)
Primary time interval, mean±SD (days)	1.1 ± 0.3	1.1 ± 0.3	1.0 ± 0.0
Total time interval, mean±SD (days)	24.8 ± 23.8	26.1 ± 26.7	27.3 ± 9.2

*TSS, transcleral suturing; IOS, intraocular suturing; n, number; SD, standard deviation; primary time interval, time interval between the occurrence of trauma and primary repairment of the globe; total time interval, time interval between the occurrence of trauma and vitrectomy*.

In this study, 91.7% of patients were injured in a uniocular manner, 8.3% were binocularly injured, while only one eye suffered from choroidal avulsion and underwent choroid suturing procedures. Of all the enrolled patients, rupture constituted the majority of the injury types (91.7%), penetrating (4.2%), and contusion (4.2%) were also observed, all patients with open globe injury involved zone III. Additionally, all patients with open-globe injuries underwent primary globe repair in 48 h (ranged from 1 to 2 days) and received vitrectomy in an average of 24.8 ± 23.8 days (ranged from 1 to 120 days), with 26.1 ± 26.7 days in the TSS group (ranged from 1 to 120 days) and 27.3 ± 9.2 days in the IOS group (ranged from 17 to 36 days).

### Intraoperative Findings

During the vitrectomy surgeries, quadrants of the avulsed choroid were assessed after the removal of vitreous hemorrhage, and the results are shown in Table 2. Complicated findings, including cornea wound or opacity, iris, and ciliary body injuries, lens conditions, retinal detachment, and proliferation were also recorded. All the patients underwent 20-gauge PPV; additional treatment procedures, such as temporary keratoprosthesis, phacoemulsification, and intraocular tamponade, were applied accordingly and shown in Table 2.

**Table 2:** Ocular characteristics and surgical interventions.

	**ALL**	**TSS**	**IOS**
	**(*n* = 24)**	**(*n* = 19)**	**(*n* = 5)**
Cornea wound/opacity, *n* (%)	17 (70.8%)	14 (73.7%)	3 (60.0%)
Irisdefect, *n* (%)	23 (95.8%)	18 (94.7%)	5 (100.0%)
Ciliary body defect (more than two quadrants involved), *n* (%)	13 (54.2%)	11 (57.9%)	2 (40.0%)
Hyphema, *n* (%)	21 (87.5%)	16 (84.2%)	5 (100.0%)
Lens, *n* (%)			
Extrusion	19 (79.2%)	14 (73.7%)	5 (100.0%)
Dislocation	2 (8.3%)	2 (10.5%)	0 (0%)
Subluxation	2 (8.3%)	2 (10.5%)	0 (0%)
Phakia	1 (4.2%)	1 (4.2%)	0 (0%)
Choroid avulsed extent, n (%)			
1 quadrant	7 (29.2%)	7 (36.8%)	0 (0%)
2 quadrants	17 (70.8%)	12 (63.2%)	5 (100.0%)
Severe intraocular hemorrhage, n (%)	24 (100.0%)	19 (100.0%)	5(100.0%)
Retina, n (%)			
Partial RD	5 (20.8%)	5 (26.3%)	0 (0%)
Total RD	5 (20.8%)	4 (21.1%)	1 (20.0%)
F-RD	14 (58.3%)	10 (52.6%)	4 (80.0%)
PVR, *n* (%)	14 (58.3%)	10 (52.6%)	4 (80.0%)
Surgical interventions, *n* (%)^a^			
Temporary keratoprosthesis	8 (33.3%)	5 (26.3%)	3 (60.0%)
Lensectomy	4 (16.7%)	4 (21.1%)	0 (0%)
Unfold of F-RD	14 (58.3%)	10 (52.6%)	4 (80.0%)
Retinotomy/retinectomy	20 (83.3%)	15 (78.9%)	5 (100.0%)
Membrane peeling	11 (45.8%)	8 (42.1%)	3 (60.0%)
C3F8	1 (4.2%)	1 (5.3%)	0 (0%)
SO	23 (95.8%)	18 (94.7%)	5 (100.0%)

*n, number; RD, retinal detachment; F-RD, closed funnel retinal detachment; SO, silicone oil*.

a *All cases underwent 20-gauge PPV; laser and perfluorocarbon were used in all*.

### Anatomical Rehabilitation and Postoperative Interventions

Reattachment of the avulsed choroid was observed in twenty-two patients (91.7%) after choroid suturing throughout the follow-ups; the other two cases without reattachment were both from the TSS group, who were found to be complicated with silicone oil migration into the suprachoroidal space at 3 months after vitrectomy. Both patients further underwent drainage of the suprachoroidal silicone oil and scleral buckling to block the remaining connection between the vitreous and suprachoroidal cavity, and at the last follow-up, the choroids were attached. Retinal reattachment was accomplished in all patients 1 day after the surgery, while partial retinal detachment due to postoperative PVR was found in one case in the TSS group at 1 month, revision vitrectomy was performed, and at the last follow-up, all patients were found with retina attached. At the last visit, four eyes (16.7%) were silicone oil-free, including one eye with primary C3F8 tamponade. Two eyes received IOL suspension. Meanwhile, 83.3% (20/24) of the eyes were silicone oil-dependent, but none of the eyes developed atrophia bulbi.

### Functional Rehabilitation at Each Follow-Up

**Figure 3:**
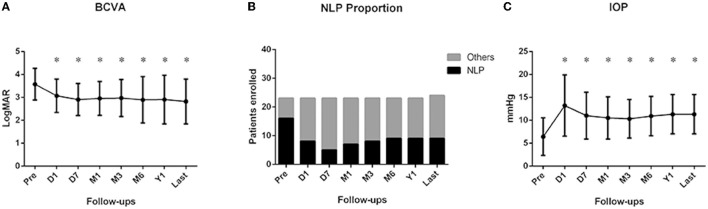
Changes of best-corrected visual acuity (BCVA), intraocular pressure (IOP), and proportion of NLP at each follow-up. **(A)** Compared with the preoperative BCVA, a significant improvement in LogMAR BCVA was observed at each follow-up after vitrectomy (all with *p* < 0.05), **(B)** Proportion of NLP was observed to be reduced from before surgery at each follow-up. **(C)** Compared with the preoperation, IOP at each postoperative follow-up was significantly increased (all with *p* < 0.05). Pre: preoperation; D1: 1 day after vitrectomy; D7: 1 week after vitrectomy; M1: 1 month after vitrectomy; M3: 3 months after vitrectomy; M6: 6 months after vitrectomy; Y1: 1 year after vitrectomy; last: last follow-up. *p < 0.05 compared with preoperation.

Figure 3A shows the BCVA before the surgery and at each follow-up in all patients. Since one patient in the TSS group aged 5, at the time of trauma, could not cooperate at the sight examination, we only recorded the BCVA of this patient at the last follow-up. Overall, compared with the preoperative BCVA (3.57 ± 0.69), a significant improvement on LogMAR BCVA was observed at each follow-up after vitrectomy (3.07 ± 0.73 at Day 1, 2.90 ± 0.70 at Day 7, 2.95 ± 0.74 at Month 1, 2.97 ± 0.81 at Month 3, 2.89 ± 1.01 at Month 6, and 2.90 ± 1.06 at Year 1, and 2.82 ± 0.98 at the last visit, all with *p* < 0.05). A small fluctuation was observed during follow-ups; however, there were no significant differences among each follow-up (all with *p* > 0.05). The proportion of NLP was also observed to be reduced (Figure 3B) from 69.6% (16/23) before surgery to 34.8% (8/23) at Day 1, 21.7% (5/23) at Day 7, 30.4% (7/23) at Month 1, 34.8% (8/23) at Month 3, 39.1% (9/23) at Month 6, 39.1% (9/23) at Year 1, and, eventually, 37.5% (9/24) at the last follow-up.

A significant elevation of IOP after vitrectomy was also observed (shown in Figure 3C). Compared with the preoperation (6.4 ± 4.1 mmHg), IOP at each postoperative follow-up, including Day 1 (13.2 ± 6.7 mmHg), Day 7 (11. ± 5.1 mmHg), Month 1 (10.5 ± 4.6 mmHg), Month 3 (10.3 ± 4.2 mmHg), Month 6 (10.9 ± 4.3 mmHg), Year 1 (11.3 ± 4.3 mmHg), and the last follow-up (11.3 ± 4.3 mmHg), was significantly increased (all with *p* < 0.05). Mean IOP at follow-ups after 1 month stayed rather stable, which indicates a long-term stable state in the patients undergoing choroidal suturing.

## Discussion

Choroid avulsion was one of the most severe traumatic conditions, which can lead to atrophia bulbi without treatment. The mechanism of choroidal avulsion has not been elucidated before. We speculate that the expulsive suprachoroidal hemorrhage leads to the separation of the choroid from the sclera first, and then the sudden increase of pressure and the shearing force in the suprachoroidal space cause the discontinuity of the detached choroid or sometimes, choroid and ciliary body disruption at the scleral spur as a whole unit, releasing the pressure in the suprachoroidal space by connection with the vitreous cavity. Meanwhile, external contusion or compression forces on the injured globe may further exacerbate the extent of the avulsion.

Due to the different histological compositions between choroid and retina, the avulsed choroid is less compliant than the detached retina to be reattached by the routine methods we use to treat retinal detachment. Very few surgical treatments have been attempted to repair choroidal avulsion. Jiang YR et al. reported a case in which medical fibrin glue was injected into the suprachoroidal cavity before the injection of the silicone oil, and the long-term outcome showed the retina and choroid were well attached (6). However, the fibrin glue can only be applied after fluid-air exchange in the non-water operation environment, which limited its use because, in many cases, the avulsed choroid should be repaired before the retina can be reattached. Meanwhile, Ma J et al. reported a suturing method in which a full-thickness scleral incision as long as the extension of the avulsion was made at the equator, and the avulsed choroid was incarcerated into the scleral incision and sutured together. They found 76.2% of eyes reached a complete choroid reattachment at 1 month after the surgery (7). Nevertheless, this suturing technique might lead to uveal exposure, which may further cause sympathetic ophthalmia. Besides, a full-thickness scleral incision at the equator may disrupt the integrity of the eyewall. Therefore, the current surgical procedures for choroidal avulsion all have their limitations.

In this study, we explored novel mattress suturing methods to repair the avulsed choroid. In both TSS and IOS, the avulsed choroid was refixed to the inner surface of the sclera. Our results indicated that these therapies had good effects on the restoration of ocular structures, with 91.7% of cases (22/24) accomplishing a successful choroid reattachment after primary vitrectomy and choroid suturing, and 8.3% (2/24) acquiring choroidal reattachment with secondary scleral buckling. On the other hand, reattachment of the choroid increased the rate of retinal attachment (95.8%); only one patient was complicated with PVR and partial retinal detachment at 1 month after the surgery, and the second vitrectomy accomplished the reattachment of retina, and at the last follow-up, all patients were observed with the full attached retina.

Visual improvement was directly correlated with the reattachment of the retina and choroid, with a significant improvement on LogMAR BCVA from 3.57 ± 0.69 before surgery to 2.82 ± 0.98 at the last follow-up (*p* < 0.05), and the proportion of NLP was also reduced from 69.6 to 37.5%. Eyes that were still NLP at the last follow-up might be owing to the ischemic injuries of the optic nerve, since the wide avulsion of short posterior ciliary arteries would shut the blood supply from its branches to the optic nerve (12).

Meanwhile, the IOP of the injured eye significantly increased from 6.4 ± 4.1 mmHg preoperatively to 11.9 ± 5.1 mmHg at the last visit (*p* < 0.05). We assume that suturing of the choroid contributes to the postoperative improvement of IOP, owing to the blockage of the abnormal passage between the vitreous cavity and the suprachoroidal cavity. However, since there was no control group in this study and silicone oil was not finally removed in twenty eyes, and the scleral buckle was also applied in two eyes, we cannot estimate exactly how beneficial the suturing techniques have on the maintenance of IOP. But, based on our previous experience, if choroidal avulsion is left untreated, phthisis will ensue despite silicone oil tamponade. Moreover, suturing of the choroid can only prevent aqueous humor passage through the suprachoroidal space but has little effect on aqueous humor production, unless the ciliary body is detached along with the choroid as a whole. In this series, therefore, twenty patients (83.3%) were silicone oil-dependent at the last visit. Dysfunction of the ciliary body complicated with severe ciliary body injury should be the main reason for silicone oil dependence in these eyes.

In this study, among the two techniques, IOS was more innovative. To our knowledge, we were the first to report putting the needle into the vitreous cavity to suture the choroid. This technique was chosen if the anterior part of the avulsed choroid was missing and/or the choroid was highly detached, making the suturing unreachable from the outside of the eye. Regarding this technique, several issues need to be noted here. First, when performing IOS, after the needle passes through the full-thickness choroid, lamellar sclera, and through the choroid again, it is advisable to cut the suture at the needle side and take the needle out of the eye before a knot is made, which decreases sliding of the suture through the choroid and minimizes the cutting effect on the choroid, as well as avoids additional damage to the intraocular tissue by the sharp needle. We suggest that a new needle with a suture is used for each stitch. Second, the blood circulation in the choroid is severely affected after avulsion occurs; therefore, suturing the avulsed choroid seldom causes bleeding, and, if it does, it is controllable with the endo-diathermy. Third, although the suture used in IOS was absorbable, we did not find postoperative suture failure in our cases. The avulsion did not recur after the suture was absorbed, implying that adhesion between choroid and sclera was generated, which assured the long-term attachment of the choroid. Last, although our results showed that intraocular suturing of the avulsed choroid was technically feasible, it takes experience and proficiency to perform this technique. Currently, there are no surgical instruments specifically designed for this suturing technique. The average operating time of vitrectomy combined with IOS in trauma cases is relatively long (~4 h).

There were also some limitations of this study: firstly, due to the relatively low incidence of choroidal avulsion, the number of the enrolled cases were limited, especially for the IOS group, and we did not analyze the influence factors of the prognosis, nor the difference between groups; long-term enrollment of patients is required in the future. Secondly, since some of the patients were from remote areas across China other than our city, part of the follow-up examinations was performed at the local hospital, and the follow-up information was acquired by a telephone interview, which might lead to some extent of information bias, which we should be aware of when interpreting our results.

## Conclusions

In conclusion, the current study explored two different suturing methods in the treatment of choroidal avulsion, which is one of the most severe ocular traumatic conditions. The follow-up results presented a high retinal and choroidal reattachment rate and significant improvements for the visual function and IOP stabilization, demonstrating these techniques may provide a much better prognosis for such severely injured eyes which otherwise cannot be preserved.

## Data Availability Statement

The raw data supporting the conclusions of this article will be made available by the authors, without undue reservation.

## Ethics Statement

The studies involving human participants were reviewed and approved by Human Research and Ethics Committee of Peking University Third Hospital. Written informed consent to participate in this study was provided by the participants' legal guardian/next of kin.

## Author Contributions

ZM and HC supervised the project. JY and HC wrote the manuscript. JY and KF conducted the statistical analysis. CW, HC, XF, and ZM collected the data. All the authors contributed to the article and approved the submitted version.

## Conflict of Interest

The authors declare that the research was conducted in the absence of any commercial or financial relationships that could be construed as a potential conflict of interest.

## Publisher's Note

All claims expressed in this article are solely those of the authors and do not necessarily represent those of their affiliated organizations, or those of the publisher, the editors and the reviewers. Any product that may be evaluated in this article, or claim that may be made by its manufacturer, is not guaranteed or endorsed by the publisher.
